# *In silico* gene expression analysis reveals glycolysis and acetate anaplerosis in *IDH1* wild-type glioma and lactate and glutamate anaplerosis in *IDH1*-mutated glioma

**DOI:** 10.18632/oncotarget.17106

**Published:** 2017-04-13

**Authors:** Mohammed Khurshed, Remco J. Molenaar, Krissie Lenting, William P. Leenders, Cornelis J.F. van Noorden

**Affiliations:** ^1^ Department of Medical Biology, Academic Medical Center, University of Amsterdam, 1105 AZ Amsterdam, The Netherlands; ^2^ Department of Pathology, Radboudumc, 6500 HB Nijmegen, The Netherlands

**Keywords:** glioma, metabolism, isocitrate dehydrogenase 1, glycolysis, glutamate

## Abstract

Hotspot mutations in isocitrate dehydrogenase 1 (*IDH1*) initiate low-grade glioma and secondary glioblastoma and induce a neomorphic activity that converts α-ketoglutarate (α-KG) to the oncometabolite *D*-2-hydroxyglutarate (*D*-2-HG). It causes metabolic rewiring that is not fully understood. We investigated the effects of IDH1 mutations (*IDH1*^MUT^) on expression of genes that encode for metabolic enzymes by data mining The Cancer Genome Atlas. We analyzed 112 *IDH1* wild-type (*IDH1*^WT^) versus 399 *IDH1*^MUT^ low-grade glioma and 157 *IDH1*^WT^ versus 9 *IDH1*^MUT^ glioblastoma samples. In both glioma types, *IDH1*^WT^ was associated with high expression levels of genes encoding enzymes that are involved in glycolysis and acetate anaplerosis, whereas *IDH1*^MUT^ glioma overexpress genes encoding enzymes that are involved in the oxidative tricarboxylic acid (TCA) cycle. *In vitro*, we observed that *IDH1*^MUT^ cancer cells have a higher basal respiration compared to *IDH1*^WT^ cancer cells and inhibition of the *IDH1*^MUT^ shifts the metabolism by decreasing oxygen consumption and increasing glycolysis. Our findings indicate that *IDH1*^WT^ glioma have a typical Warburg phenotype whereas in *IDH1*^MUT^ glioma the TCA cycle, rather than glycolytic lactate production, is the predominant metabolic pathway. Our data further suggest that the TCA in *IDH1*^MUT^ glioma is driven by lactate and glutamate anaplerosis to facilitate production of α-KG, and ultimately *D*-2-HG. This metabolic rewiring may be a basis for novel therapies for *IDH1*^MUT^ and *IDH1*^WT^ glioma.

## INTRODUCTION

Diffuse gliomas are infiltrative neoplasms that arise in the cerebral hemispheres of adults and are graded as WHO II-IV based on histopathological characteristics. Grade II and III are low-grade gliomas (LGG) of which a subset may progress to secondary glioblastoma (WHO grade IV), whereas others may remain relatively stable for longer periods of time. In contrast to this gradual progression of malignancy, the majority of glioblastomas arise *de novo*, and are then called primary glioblastoma. Complete surgical resection of diffuse glioma is impossible due to their highly invasive nature and without exception residual tumor is a source of recurrence and malignant progression. Median overall survival of LGG ranges widely from 3 to 15 years and patients with *de novo* primary glioblastoma, the most common and aggressive primary brain tumor, have a median overall survival of 16 months. Despite all efforts to therapeutically target various pathways that are involved in glioma progression, significant improvement in survival has not been achieved since 2005, when temozolomide was added to irradiation as standard therapy [1, 2].

A subset of gliomas displays increased sensitivity towards chemo- and radiotherapy with prolonged median overall patient survival. Recently, it was demonstrated that these characteristics are strongly associated with mutations in the metabolic enzyme isocitrate dehydrogenase (IDH) [3]. Hotspot mutations in *IDH1* and, less frequently, *IDH2* occur in 80% of WHO grade II-III and secondary WHO grade IV gliomas and are ancestral events in the formation of these neoplasms [4, 5]. In addition to glioma, *IDH1/2* mutations (*IDH1/2*^MUT^) occur in substantial percentages in various other tumor types, such as acute myeloid leukemia (20–40%), chondrosarcoma (60%), intrahepatic cholangiocarcinoma (20%) and melanoma (5–10%) [6].

IDH1 and IDH2 are homodimeric enzymes that reversibly convert isocitrate to α-ketoglutarate (α-KG) with concomitant reduction of NADP^+^ to NADPH in the cytoplasm and mitochondria, respectively. The mutations lead to a neomorphic activity of the enzyme that converts α-KG into the oncometabolite *D*-2-hydroxyglutarate (*D*-2-HG). *D*-2-HG competitively inhibits α-KG-dependent enzymes [7, 8], including histone demethylases and 5-methylcytosine hydroxylases that are essential for epigenetic regulation of gene expression, including that of metabolic genes [9–13]. *IDH1/2*^MUT^ changes cellular metabolism via 4 mechanisms: 1) loss of wild-type IDH1/2 (IDH1/2^WT^) function that affects carbohydrate and NADP^+^/NADPH metabolism; 2) accumulation of *D*-2-HG that further restricts the activity of various enzymes such as α-ketoglutarate dehydrogenase (α-KGDH), succinate dehydrogenase (SDH) and complex IV of the mitochondrial electron transport chain, 3) epigenetic effects of *D*-2-HG on expression of genes involved in metabolism and 4) increased degradation of the hypoxia-response transcription factor HIF-1α, a major inducer of expression of genes involved in glycolysis [3, 6, 14, 15].

Reprogramming of cellular metabolism is one of the hallmarks of cancer [16]. It is generally assumed that malignant cells use pyruvate for lactate production instead of fluxing it into acetyl-CoA in the TCA cycle, even in the presence of oxygen [17]. This so-called Warburg effect is an accepted metabolic phenotype in cancer that allows rapid generation of biosynthetic intermediates at the expense of less efficient ATP production. As a result, cancer cells overexpress glucose transporters to compensate for their ATP demand via an abnormally high rate of glucose uptake. Presumably, the composition and availability of metabolites in the microenvironment, for example in the adult brain glucose, glutamate, glutamine and acetate, dictates which metabolites are the predominant carbon sources for cancer cells. These metabolites can all serve as precursors for macromolecules such as proteins, nucleotides and lipids [18, 19].

It has been reported that glioma cells have a higher rate of aerobic glycolysis than healthy brain tissue, and this is more pronounced in glioblastoma than in LGG [20, 21]. However, these observations may be biased because most analyses of glioblastoma tissue use resection material that likely contains angiogenic, hypoxic and necrotic regions. Metabolism in these areas may well differ from that in peripheral invasive regions that presumably do not experience hypoxia. Talasila et al. [22] reported recently that the centre the tumor centre of glioblastoma is particularly glycolytic, in line with the presence of perinecrotic hypoxia, and we previously observed similar metabolic phenotypes in preclinical orthopic xenograft models [23]. However, how metabolic pathways in glioma are affected by *IDH1*^MUT^ is not completely clear.

In order to evaluate the metabolic rewiring associated with *IDH1*^MUT^, we performed an *in silico* analysis of the transcripts that encode for metabolic enzymes and substrate transporters. These gene expression data were correlated with *IDH1* mutational status in 677 LGG and glioblastoma samples derived from The Cancer Genome Atlas (TCGA). This study provides a comprehensive overview of the expression of key glycolytic, TCA cycle and glucose/lactate/glutamate/acetate anaplerosis enzymes in *IDH1*^MUT^ glioma versus *IDH1*^WT^ glioma.

## RESULTS

We determined differences in expression levels of metabolic enzymes in *IDH1*^WT^ versus *IDH1*^MUT^ glioma to increase our understanding of the metabolism of these two clinically very distinct types of gliomas. We included 399 *IDH1*^MUT^ and 112 *IDH1*^WT^ LGG (WHO stage II: 49% and WHO stage III: 51%) and 157 *IDH1*^WT^ and 9 *IDH1*^MUT^ WHO stage IV glioblastoma samples. The ratios of IDH1-mutated to IDH1 wild-type tumors are in agreement with mutation frequencies from earlier reports of TCGA glioma data and other datasets. In line with previous reports [4, 24, 25], our dataset shows an approximately 3-fold prolonged survival of *IDH1*^MUT^ LGG and glioblastoma patients compared to *IDH1*^WT^ LGG and glioblastoma patients (Supplementary Figure 1).

### Expression of GLUT3 and glucose-metabolizing enzymes is low in *IDH1*^MUT^ compared to *IDH1*^WT^ glioma

**Figure 1:**
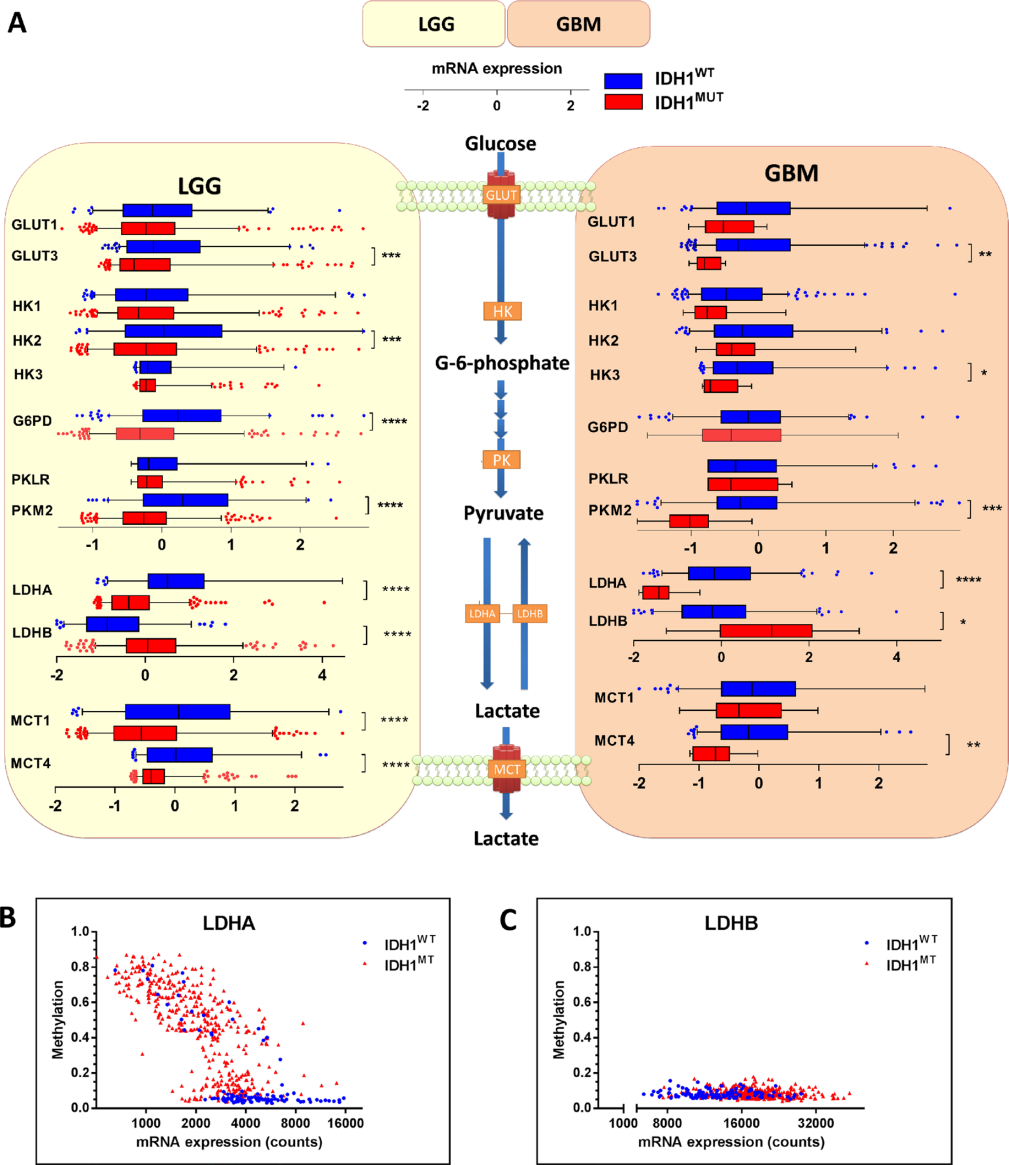
mRNA expression levels of enzymes involved in glucose metabolism in *IDH1*^WT^ versus *IDH1*^MUT^ glioma *IDH1*^WT^ glioma show higher expression levels of genes that are typically upregulated in glycolytic cancer cells. (**A**) Analysis of gene expression levels of metabolic enzymes involved in transport and metabolism of glucose in *IDH1*^WT^ (*n* = 399) and *IDH1*^MUT^ (*n* = 112) LGG and *IDH1*^WT^ (*n* = 157) and *IDH1*^MUT^ (*n* = 9) glioblastoma samples obtained from the TCGA database. Relative mRNA expression levels are shown for *IDH1*^WT^ (blue) and *IDH1*^MUT^ (red). (**B**) *LDHA* and (**C**) LDHB expression and methylation according to *IDH1*^MUT^ status (blue: *IDH1*^WT^, red: *IDH1*^MUT^). Abbreviations: GLUT, glucose transporter; HK, hexokinase; G6PD, glucose-6-phosphate dehydrogenase; PK, pyruvate kinase; LDH, lactate dehydrogenase.

To investigate the first rate-limiting step of cellular glucose metabolism, i.e. transport of glucose across the plasma membrane, we examined expression levels of *SLC2A1* and *SLC2A3*, the genes encoding for glucose transporters (GLUT1 and GLUT3). GLUT3 expression levels were low in *IDH1*^MUT^ glioma when compared with *IDH1*^WT^ glioma, whereas GLUT1 expression did not differ between *IDH1*^MUT^ and *IDH1*^WT^ glioma (Figure 1A). The rate-limiting, irreversible and glycolysis-related enzymes hexokinase (HK2 and HK3) and pyruvate kinase (PKM2), but not HK1 or the pyruvate kinase isoform PKLR, were also expressed at lower levels in *IDH1*^MUT^ glioma compared with *IDH1*^WT^ glioma (Figure 1). High expression of HK2 was significant in *IDH1*^WT^ LGG, whereas HK3 expression was significant higher in *IDH1*^WT^ glioblastoma. The rate-limiting enzyme of the pentose-phosphate pathway, glucose-6-phosphate dehydrogenase (G6PD) was expressed at higher levels in *IDH1*^WT^ glioma, but only in LGG and not glioblastoma. These results suggest that *IDH1*^WT^ glioma depend more on glycolysis than *IDH1*^MUT^ glioma.

### *IDH1*^WT^ glioma express Warburg-effect genes, whereas *IDH1*^MUT^ glioma express TCA cycle genes

The relative activities of lactate fermentation in the glycolysis and the TCA cycle are controlled by two sets of enymes that determine the fate of pyruvate: pyruvate dehydrogenase (PDH) and the lactate dehydrogenases (LDHA and LDHB, where LDHA catalyzes conversion of pyruvate into lactate and LDHB catalyzes the reverse reaction). In *IDH1*^WT^ glioma, we observed a striking overexpression of LDHA relative to *IDH1*^MUT^ glioma (Figure 1A). Lower expression levels of LDHA in *IDH1*^MUT^ glioma were associated with hypermethylation of its promoter (Figure 1B). Conversely, *IDH1*^MUT^ glioma showed higher LDHB expression levels than *IDH1*^WT^ glioma. To maintain pH homeostasis, cancer cells up-regulate transporters such as the monocarboxylate transporters 1 and 4 (MCT1 and MCT4) for secretion of lactate [26, 27]. In *IDH1*^WT^ glioma, we observed higher expression of MCT1 and MCT4 as compared to *IDH1*^MUT^ glioma, suggesting increased transport of lactate in *IDH1*^WT^ glioma. These results indicate that *IDH1*^WT^ gliomas catabolize glucose in the glycolytic pathway with lactate as end product whereas in *IDH1*^MUT^ glioma lactate is converted into pyruvate.

**Figure 2:**
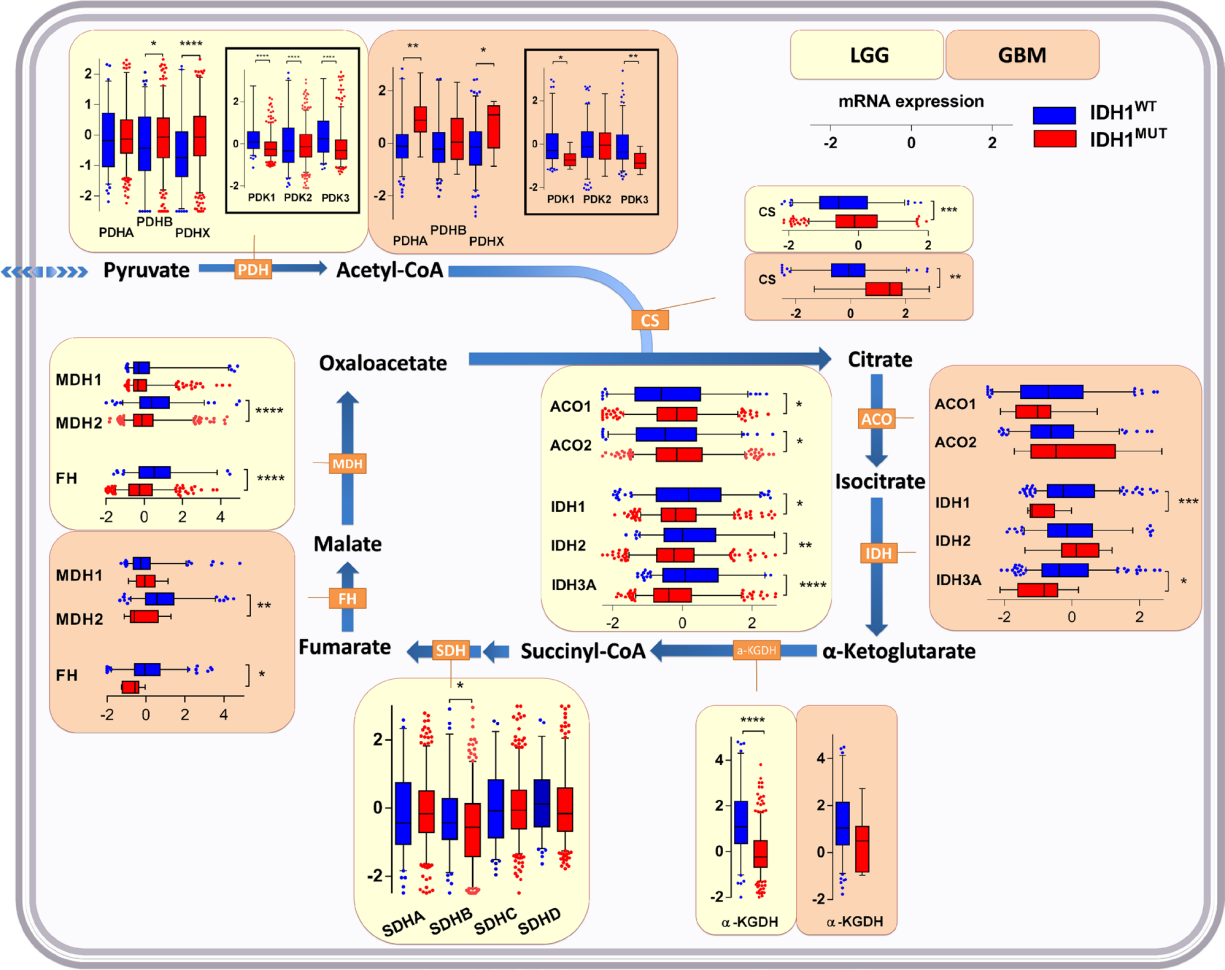
Expression levels of genes of enzymes involved in the TCA cycle in *IDH1*^WT^ versus *IDH1*^MUT^ glioma Increased TCA upstream of isocitrate, but decreased metabolism downstream of isocitrate in *IDH1*^MUT^ glioma as indicated by gene expression levels of enzymes involved in the TCA cycle in *IDH1*^WT^ (*n* = 399) and *IDH1*^MUT^ (*n* = 112) LGG and *IDH*^1WT^ (*n* = 157) and *IDH1*^MUT^ (*n* = 9) glioblastoma. Relative mRNA expression levels are shown for *IDH1*^WT^ (blue) and *IDH1*^MUT^ (red). Abbreviations: PDH, pyruvate dehydrogenase; PDK, pyruvate dehydrogenase kinase; CS, citrate synthase; ACO, aconitase; IDH, isocitrate dehydrogenase; a-KGDH, α-ketoglutarate dehydrogenase; SDH, succinate dehydrogenase; FH, fumarate hydratase; MDH, malate dehydrogenase.

Conversion of pyruvate into acetyl-CoA for its entry into the TCA is accomplished by the pyruvate dehydrogenase (PDH) complex, the activity of which is controlled by pyruvate dehydrogenase kinase (PDK; isoforms PDK1, PDK2 and PDK3). PDK inactivates PDH via phosphorylation. PDH is a heterotrimeric enzyme complex composed of subunits PDHA and PDHB, forming the catalytic PDH component PDH E1, and PDHX, a non-catalytic regulatory subunit. In *IDH1*^WT^ glioma, we observed higher expression of regulator genes PDK1 and PDK3 as compared to *IDH1*^MUT^ glioma. On the other hand, *IDH1*^MUT^ glioma showed higher expression levels of PDHA (in glioblastoma), PDHB (in LGG) and PDHX as compared to *IDH1*^WT^glioma (Figure 2). Combined with the aforementioned glycolysis data these results suggest that in contrast to *IDH1*^WT^ glioma, *IDH1*^MUT^ glioma actively prevents the generation of lactate by silencing LDHA and maintaining high expression of LDHB, PDHA/B and PDHX. These conditions ensure that pyruvate is maximally used for TCA entry.

### *IDH1*^MUT^ glioma have increased expression of TCA genes upstream of isocitrate and decreased expression of TCA genes downstream of isocitrate

Intermediate metabolites of glycolysis upstream of pyruvate can be shunted into macromolecular biosynthesis pathways without stalling glycolysis itself. In contrast, the TCA cycle must be continuously running and be completed to function optimally: generation of citrate by citrate synthase (CS) requires pyruvate-derived acetyl-CoA, the principal fuel of mitochondria, and the last TCA cycle metabolite (oxaloacetate). Mitochondrial products of a full TCA cycle are ATP and CO_2_. The lack of glycolysis in *IDH1*^MUT^ gliomas suggests that these cancers apply alternative metabolic pathways that ensure sufficient energy and building blocks for macromolecular biosynthesis. To investigate this in detail, we examined expression levels of TCA-related enzymes in *IDH1*^MUT^ and *IDH1*^WT^ gliomas.

**Figure 3:**
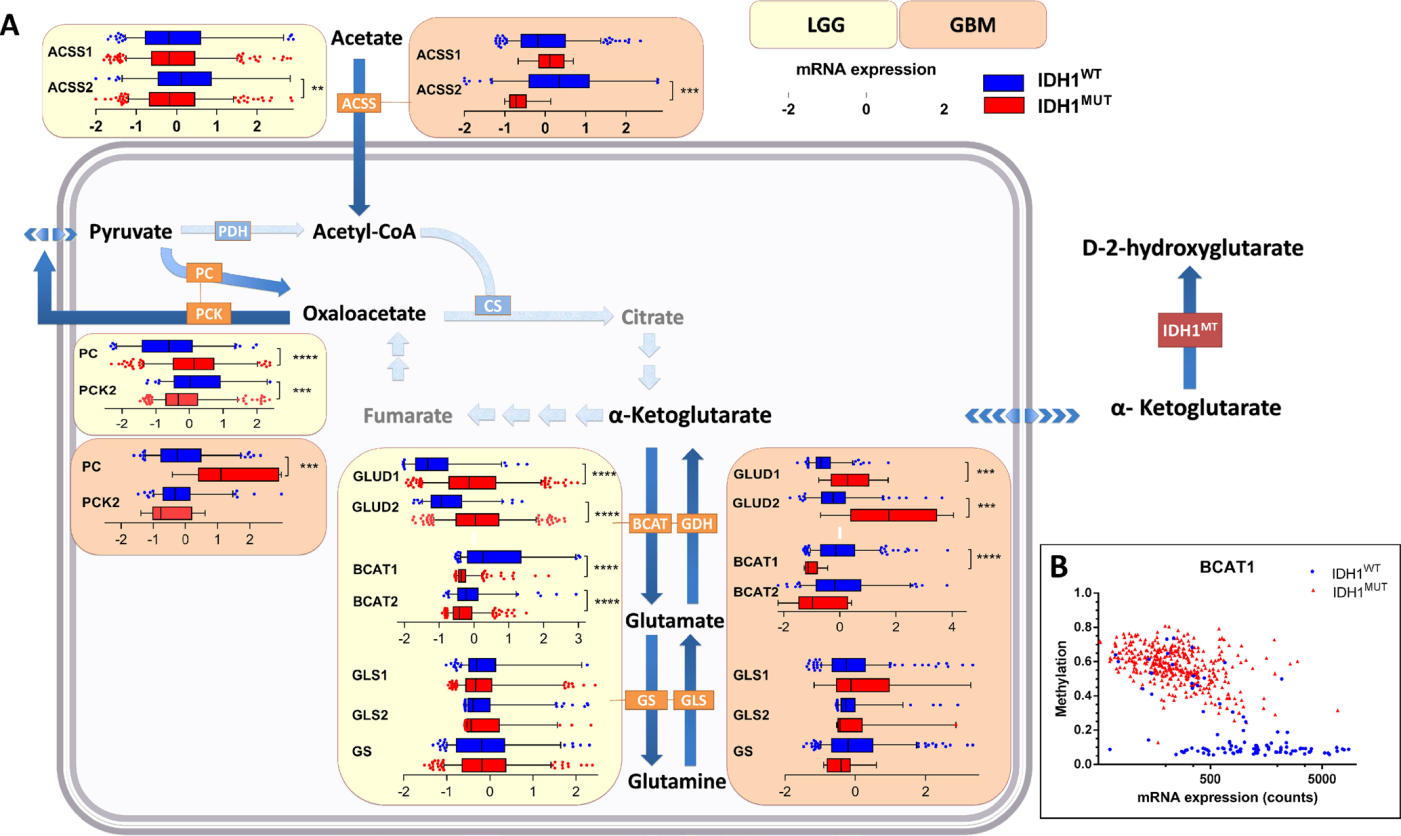
Contribution of glucose/glutamine/glutamate anaplerosis and acetate influx to TCA cycle metabolism in *IDH1*^WT^ versus *IDH1*^MUT^ glioma Increased glucose/glutamate anaplerosis in *IDH1*^MUT^ glioma and high contribution of acetate in *IDH*^WT^ glioma metabolism. (**A**) Gene expression levels of metabolic enzymes involved in glucose anaplerosis, glutaminolysis and acetate metabolism in *IDH1*^WT^ (*n* = 399) and *IDH1*^MUT^ (*n* = 112) LGG and *IDH1*^WT^ (*n* = 157) and *IDH1*^MUT^ (*n* = 9) glioblastoma. Relative mRNA expression levels are shown for *IDH1*^WT^ (blue) and *IDH1*^MUT^ (red). (**B**) *BCAT1* expression and methylation according to *IDH1*^MUT^ status (blue: *IDH1*^WT^, red: *IDH1*^MUT^). Abbreviations: PC, pyruvate carboxylase; GLUD, glutamate dehydrogenase; GLS, glutaminase; BCAT, branched-chain amino acid transferase; GS, glutamine synthetase; ACSS, acetyl-CoA synthetase.

CS and aconitase (ACO1/2), enzymes that function upstream of citrate oxidation, were expressed at higher levels in *IDH1*^MUT^ glioma than in *IDH1*^WT^ glioma (Figure 2). In contrast, expression levels of enzymes involved in downstream metabolism of isocitrate, including IDH1, IDH2 and IDH3A, α-KGDH, succinate dehydrogenase (SDHB), fumarate hydratase (FH) and malate dehydrogenase (MDH2), were remarkably lower in *IDH1*^MUT^ glioma versus *IDH1*^WT^ glioma. These results suggest that *IDH1*^MUT^ glioma have an imbalanced TCA, with increased enzyme activity upstream of isocitrate, and decreased TCA cycle metabolism downstream of isocitrate. The imbalance resulting from low oxaloacetate production and high pyruvate influx causes disbalance that may be problematic for efficient citrate production. As a compensatory mechanism, oxaloacetate can also be produced directly from pyruvate by the activity of pyruvate carboxylase (PC). This reaction is counteracted by phosphoenolpyruvate carboxykinase 2 (PCK2) which catalyzes the reaction of oxaloacetate to phosphoenolpyruvate. In *IDH1*^MUT^ glioma, PC levels were markedly higher than in *IDH1*^WT^ glioma, whereas PCK2 expression levels were significantly lower in *IDH1*^MUT^ glioma although differences in PCK2 levels between *IDH1*^WT^ and *IDH1*^MUT^ in glioblastoma were less apparent (Figure 3). Collectively, these results indicate increased flux of pyruvate into the TCA cycle in *IDH1*^MUT^ glioma compared to *IDH1*^WT^ counterparts.

### Higher lactate/glutamate anaplerosis in *IDH1*^MUT^ glioma and higher acetate anaplerosis in *IDH*^WT^ glioma

We have previously postulated the hypothesis that glutamate could act as anaplerotic precursor for the TCA cycle in *IDH1*^MUT^ glioma [28]. To test this assumption, we analyzed the expression levels of mRNA of enzymes that are involved in the transport and conversion of these metabolites.

Glutamine is a major source of TCA cycle metabolites via glutaminolyis, a process that converts glutamine to α-KG via two subsequent deamination steps. To investigate its contribution to α-KG replenishment in the TCA cycle, we determined mRNA expression levels of glutaminase (GLS and GLS2, converting glutamine to glutamate) and glutamate dehydrogenase (GLUD1 and GLUD2, converting glutamate to α-KG). We also investigated mRNA expression levels of branched-chain amino acid transferase (BCAT1 and BCAT2) and glutamine synthetase (GS), which catalyze the converse reaction of α-KG into glutamate and of glutamate into glutamine, respectively. In *IDH1*^MUT^ glioma, we observed strikingly higher expression levels of GLUD1/2 than in *IDH1*^WT^ glioma, whereas BCAT1/2 expression levels were much lower in *IDH1*^MUT^ gliomas than in *IDH1*^WT^ glioma (Figure 3), as has been reported previously [29]. Low BCAT1 mRNA expression levels in *IDH1*^MUT^ glioma were associated with BCAT1 promoter hypermethylation (Figure 3B).

We observed no significant differences between mRNA expression levels of GLS, GLS2 or GS between *IDH1*^WT^ glioma and *IDH1*^MUT^ glioma. These results suggest that *IDH1*^MUT^ gliomas predominantly utilize glutamate, rather than glutamine, for TCA cycle anaplerosis.

Acetate can be utilized as an alternative substrate to generate acetyl-CoA and is transferred through the plasma membrane by MCT1 and MCT4 [30]. Acetyl-CoA synthetase (ACSS1 and ACSS2) converts acetate to acetyl-CoA, which can be catabolized in the TCA cycle. In *IDH1*^WT^ glioma, ACSS2 showed an elevated mRNA expression compared to *IDH1*^MUT^ glioma (Figure 3), suggesting a higher contribution of acetate to the acetyl-CoA pool in *IDH1*^WT^ glioma. As indicated previously, higher contribution of acetate is in line with higher expression levels of MCT1 and MCT4 transporters in *IDH1*^WT^ glioma.

Taken together, we show that relative to *IDH1*^WT^ glioma, *IDH1*^MUT^ glioma use lactate and glutamate (but lesser so glutamine) for the TCA cycle, by maintaining high expression of LDHB, PC and GLUD and silencing BCAT. In contrast, in *IDH1*^WT^ glioma glycolysis is more prominent and acetate is used to fuel the TCA cycle.

### Inhibition of the *IDH1*^MUT^ decreases oxygen consumption and increases lactate acidification

**Figure 4:**
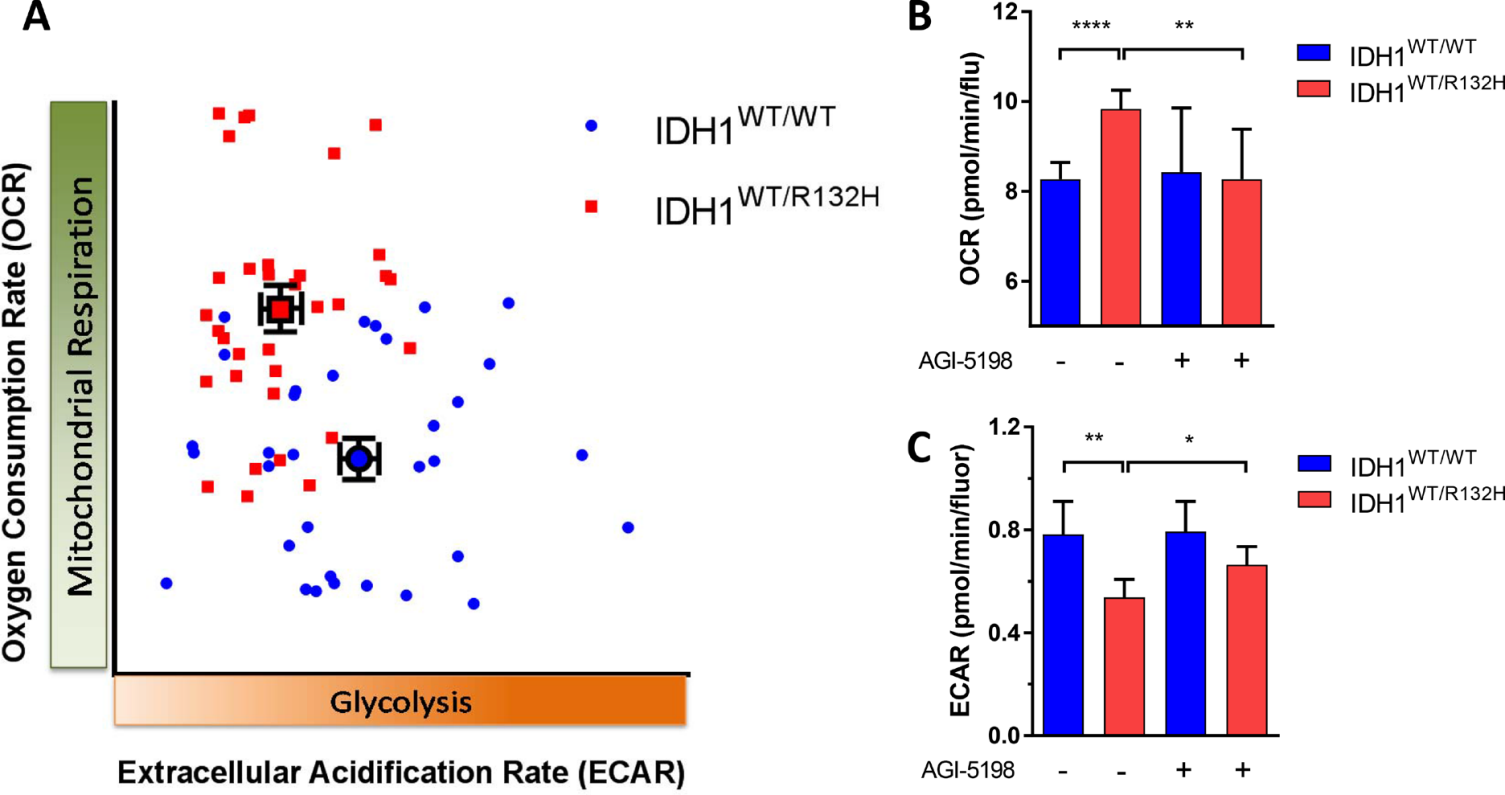
The metabolic phenotype of *IDH1*^MUT^ and *IDH1*^WT^ (**A**) Characterization of metabolic phenotype; HCT116 *IDH1*^WT/R132H^ cells (red) are dependent on OXPHOS, whereas HCT116 *IDH1*^WT/WT^ cells (blue) cells are glycolytic. (**B**) The basal oxygen consumption rate (OCR) response of the HCT116 cell lines to 10 mM glucose, 2 mM glutamine and 1.5 mM pyruvate, with or without pretreatment with the *IDH1*^MUT^ inhibiter AGI-5198 (1 μM, 14 days of incubation). (**C**) Extracellular acidification rate (ECAR) response of HCT116 cells to glucose (25 mM), with or pretreatment with of AGI-5198 (1 μM, 14 days of incubation). All data were expressed as pmol of O_2_ per minute and normalized by cell number measured by fluorochrome binding to nucleic acids. A representative experiment out of 4 is shown here, each data point represents mean ± SEM. Plots are visualized with 95% confidence intervals and significance levels are shown by (*P < 0.05), (**P < 0.01) and (****P < 0.0001).

There is a scarcity of relevant models to study metabolic effects of IDH mutations *in vitro* [31]. As an alternative we used the HCT116-IDH1^R132H^ knock in cell line and its parental counterpart to investigate whether metabolic changes in *IDH1*^R132H^ gliomas were reflected in this isogenic cell line pair too. We first measured oxygen consumption in HCT116 *IDH1*^WT/R132H^ and HCT116 *IDH1*^WT/WT^ cells in the Seahorse [32]. The basal respiration was significantly higher in HCT116 *IDH1*^WT/R132H^ cells than in HCT116 *IDH1*^WT/WT^ cells, showing that also in this system *IDH1*^MUT^ cells have higher oxygen consumption and are more dependent on OXPHOS (Figure 4). To determine whether the *IDH1*^MUT^ is causally involved in the higher respiration rates, we tested the effects of the specific IDH1^R132H^ inhibitor AGI-5198. In line with expectations after 14 days of culture in the presence of the *IDH1*^MUT^ inhibitor AGI-5198 the respiration rate of HCT116 *IDH1*^WT/R132H^ cells was significantly decreased and comparable to levels of HCT116 *IDH1*^WT/WT^ cells (Figure 4B).

In order to investigate the glycolytic activity, we measured the extracellular acidification rate (ECAR) that determines the glycolytic phenotype of cells and is linearly related to lactate production [33, 34]. In HCT116 *IDH1*^WT/R132H^ cells, we found a lower ECAR response to glucose compared to HCT116 *IDH1*^WT/WT^ cells, indicating a lower glycolytic rate in *IDH1*^MUT^ cells (Figure 4). The glycolytic capacity was also significantly lower in HCT116 *IDH1*^WT/R132H^ cells compared to HCT116 *IDH1*^WT/WT^ cells (data not shown). After 14 days incubation in the presence of AGI-5198, we measured a slight increase of ECAR in HCT116 *IDH1*^WT/R132H^ cells, indicating a shift of metabolism to glycolysis by inhibiting *IDH1*^MUT^ (Figure 4C). Taken together, we show that relative to *IDH1*^WT^ cells*, IDH1*^MUT^ cells are dependent on OXPHOS and inhibition of the *IDH1*^MUT^ decreases the oxygen consumption leading to a glycolytic phenotype.

## DISCUSSION

**Figure 5:**
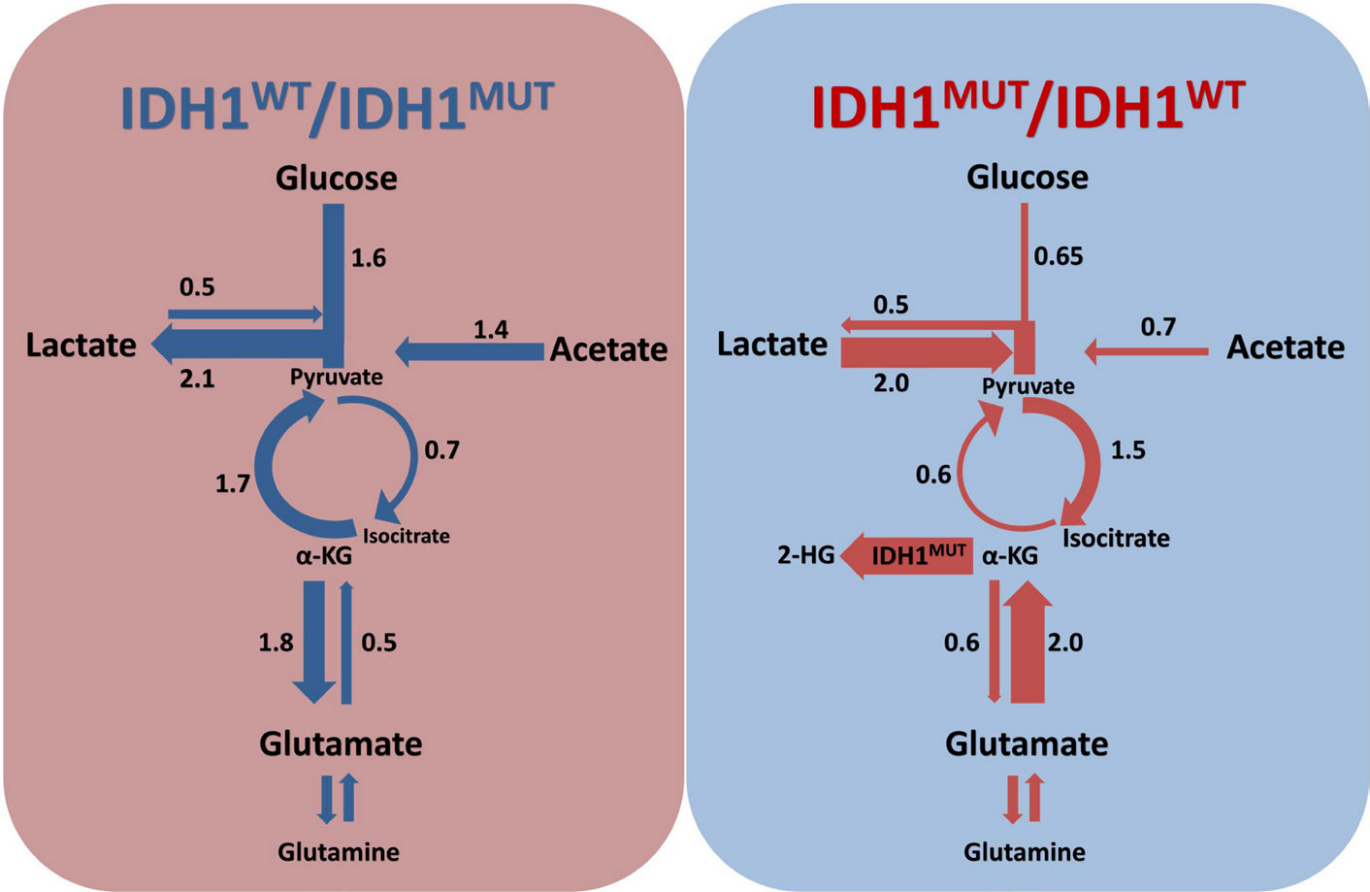
Rewiring of metabolism by *IDH1*^MUT^ *IDH1*^WT^/*IDH1*^MUT^ and *IDH1*^MUT^/*IDH1*^WT^ ratios indicate contribution of a particular pathway as calculated on the basis of gene expression levels in *IDH1*^WT^ and *IDH1*^MUT^, respectively. *IDH1*^WT^ glioma catabolize glucose in the glycolysis with lactate as end product, whereas *IDH1*^MUT^ glioma prefers production of pyruvate from lactate and use the TCA cycle to generate isocitrate. Glucose, lactate and glutamate replenish the TCA cycle in *IDH1*^MUT^ glioma, to facilitate α-KG production for consumption by IDH1^MUT^ to generate 2-HG, whereas acetate anaplerosis is important in *IDH1*^WT^ glioma.

The present study is the first to investigate the metabolic rewiring that is associated with *IDH1*^MUT^ in glioma in a comprehensive and integrated fashion. A summary of this metabolic rewiring is shown in Figure 5. We correlated the expression of key glycolytic, TCA cycle and glucose/lactate/glutamate/acetate anaplerosis enzymes with the *IDH1*^MUT^ status obtained from a large database containing data on both LGG and glioblastoma patients. We show that *IDH1*^WT^ glioma overexpress enzymes that play key roles in anaerobic glycolysis, *i.e*. genes that are typically upregulated in Warburg-like cancer cells. In contrast, expression of genes that encode for glycolytic metabolism are downregulated in *IDH1*^MUT^ glioma. Based on the axiom that gene expression levels are correlated with pathway activity, our data suggest that *IDH1*^MUT^ glioma predominantly catabolize glutamate, and to a lesser extent glucose, in the TCA cycle for α-KG production. In support of this axiom, we confirmed these observations in functional studies by measuring lactate production and oxygen consumption in HCT116 *IDH1*^WT/WT^ and HCT116 *IDH1*^WT/R132H^ cells.

Glycolysis and lactate production are considered to be the major metabolic ways of processing of glucose in cancer cells as it generates both energy and carbon for macromolecular biosynthesis, required for sustained cell proliferation [35]. In particular, GLUT3, HK2, PKM2 and LDHA play critical roles in initiating and maintaining the high glycolytic rates of rapidly proliferating cancer cells, and are associated with greater dependence on glycolysis than on OXPHOS [36–40]. Our findings are in agreement with the existence of the Warburg effect in *IDH1*^WT^ glioma, whereas our data indicate that glycolysis and lactate production occur to a lesser extent in *IDH1*^MUT^ glioma. In line with this notion, silencing of HK2 increases oxygen consumption and decreases lactate production in glioblastoma cells [41]. The elevated levels of LDHB in *IDH1*^MUT^ gliomas support the findings in previous studies that showed that *IDH1*^MUT^ glioma have reduced intracellular lactate levels compared to *IDH1*^WT^ glioma, [42, 43]. Moreover, a recent study showed that the conversion rate of pyruvate into lactate was similar in *IDH1*^MUT^ glioma and normal brain, whereas *IDH1*^WT^ glioma showed a higher lactate production than normal brain [44]. Our results also support previous reports that show that *IDH1*^MUT^ gliomas silence *LDHA* expression through hypermethylation of its promoter [43] resulting in a low LDHA/LDHB ratio compared with *IDH1*^WT^ glioma or normal brain tissue [45]. Reduced MCT expression in *IDH1*^MUT^ glioma is consistent with reduced glycolytic production of lactate as well as silencing of LDHA expression; cells that produce less lactate do not require an increase in its export and are less dependent on MCT expression.

Lactate production by *IDH1*^WT^ glioma cells has been suggested to acidify the extracellular space [46, 47] resulting in the death of surrounding normal brain cells and increasing the infiltrative potential of cancer cells [48]. Our results suggest that *IDH1*^MUT^ glioma produce and secrete less lactate than *IDH1*^WT^ glioma which may correlate with a normalized tissue pH in *IDH1*^MUT^ glioma and the less aggressive behavior of *IDH1*^MUT^ glioma as compared to *IDH1*^WT^ glioma [49].

Compared with *IDH1*^WT^ glioma, *IDH1*^MUT^ glioma showed elevated expression levels of TCA cycle genes that function upstream of isocitrate whereas expression of TCA cycle genes downstream of isocitrate were decreased. In line with these observations, a recent study reported high expression of CS and ACO2 in *IDH1*^MUT^ glioma versus *IDH1*^WT^ glioma [45]. We have shown previously that IDH enzymatic activity in *IDH1*^MUT^ glioblastoma is reduced compared to *IDH1*^WT^ glioblastoma [25]. Consistent with these findings, a previous report demonstrated increased levels of isocitrate (2.7-fold) and *D*-2-HG (28-fold) in *IDH1*^MUT^ cancer cells versus *IDH1*^WT^ cancer cells [8, 50]. In addition to decreased SDH expression in *IDH1*^MUT^ glioma, a recent study demonstrated elevated succinate levels and protein succinylation in *IDH1*^MUT^ glioma samples [51]. Taken together, the changes in gene expression levels in *IDH1*^MUT^ suggest that mitochondrial isocitrate production is maximized, providing substrate for the cytosolic IDH1^WT^/IDH1^MUT^ dimer complexes to generate α-KG and *D*-2-HG, respectively.

Due to the lack of stable glioma cell cultures carrying an endogenous *IDH1*^R132H^ allele, we used *in vitro* HCT116 colorectal carcinoma cells for functional studies. *IDH1*^MUT^ occur in 0.5% of colorectal carcinomas [52]. Because *IDH1*^R132H^ functions as a heterodimer with *IDH1*^WT^, 1:1 *IDH1*^R132H^*:IDH1*^WT^ expression in *IDH1*^WT/R132H^ HCT116 cells is more relevant than IDH1^R132H^ overexpression models that are frequently used. Previous reports have shown that HCT116 *IDH1*^MUT^ cells have reduced growth rates as compared to *IDH1*^WT^ cells under conditions of low oxygen tension suggesting that these are more dependent on OXPHOS [53].

Another major contributor to TCA metabolism in cancer cells is glutamine, which provides α-KG via the glutaminolysis pathway. Moreover, 80% of *D*-2-HG generated by heterozygous *IDH1*^WT/MUT^ HCT116 colorectal cancer cells is derived from glutamine [53, 54], rendering this an important metabolite for *D*-2-HG generation. Whereas glutamine has been suggested to be an important carbon source in several types of cancers, experiments in xenograft models revealed that glioblastoma cells take up glutamine but do not directly oxidize it in the TCA cycle [55]. Our analysis suggests an increased contribution of glutamate, rather than glutamine, to the TCA cycle in *IDH1*^MUT^ glioma, by maintaining high expression levels of GLUD1/2 and silencing or downregulation of BCAT expression. Our results support the findings of previous studies that demonstrated increased conversion of glutamate to α-KG by GLUD [45, 56] and reduced conversion of α-KG to glutamate by BCAT1 in *IDH1*^MUT^ glioma, which is in line with promoter methylation of BCAT1 in *IDH1*^MUT^ glioma [29, 57].

In line with our observations, a recent study demonstrated that *IDH1*^MUT^ overexpression in astrocytes results in increased PC expression and increased fractional flux through PC, suggesting that direct oxaloacetate production from pyruvate is critical for *IDH1*^MUT^ cancer cells to maintain TCA activity [58]. Furthermore, metabolic flux studies in *IDH1*^MUT^ glioma models with [1–^13^C]-glucose showed that 20% of 2-HG was derived from glucose, suggesting that glucose is a less relevant donor of carbon for 2-HG production than glutamate [54]. On the other hand, we observed a high expression of PCK2 that shuttles oxaloacetate into glycolysis suggesting increased contribution from the TCA cycle to glycolysis in *IDH1*^WT^glioma. In line with this observation, a study also reported high expression of PCK2 in *IDH1*^WT^ glioma versus *IDH1*^MUT^ glioma [45].

Finally, acetate metabolism can replenish the TCA cycle at the level of acetyl-CoA and this is an important anaplerosis pathway in the majority of glioblastoma [55]. Our analyses suggest that this phenomenon is more important in *IDH1*^WT^ glioma than in *IDH1*^MUT^ glioma. Mashimo *et al*. did not stratify their glioblastoma cohort based on *IDH1*^MUT^ status but demonstrated higher expression of ACSS2 in glioblastoma than in grade II and III glioma and high ACSS2 expression in LGG patients with poor survival as opposed to low ACSS2 expression in LGG patients with prolonged survival. This resonates with the high frequency of *IDH1*^MUT^ in LGG cohorts and clarifies the observed association of ACSS2 expression and LGG patient survival, potentially reflecting the differences in ACSS2 expression and patient survival between *IDH1*^MUT^ and *IDH1*^WT^ glioma.

Our transcriptome-wide approach study was performed on a large number of LGG and glioblastoma samples derived from TCGA. These data have been analyzed previously in an effort to improve the pathological classification of glioma. It was proposed to categorize LGG in 3 molecular classes, based on *IDH1, 1p/19q* and *TP53* status, instead of histology [59]. In addition, *IDH1*^WT^ LGG appeared to be molecularly and clinically more similar to *IDH1*^WT^ glioblastoma than to *IDH1*^MUT^ LGG. Our studies support these conclusions in more detail, based on the profound similarities in metabolic transcript profiles both in *IDH1*^WT^ gliomas and in *IDH1*^MUT^ gliomas, irrespective of WHO grade. Of note, our data are confirmed by independent studies showing lower activity of HK, SDH and ACSS2 and higher activity of CS in LGG compared to glioblastoma [41, 55, 60]. Considering the fact that *IDH1*^MUT^ is more prevalent in LGG than in glioblastoma (80% versus 12%), this suggests that *IDH1*^MUT^ is an important determinator in histological analyses and that metabolic analyses should be stratified by *IDH1*^MUT^ status and not histology.

These insights are relevant for rational development of metabolic anti-glioma therapies, because our data predict that inhibitors of glycolysis, such as HK2/3 and PKM2 [61, 62] may be the most promising for *IDH1*^WT^ glioma, whereas inhibitors of the TCA cycle and the glutamatolysis pathway such as metformin, chloroquine and epigallocatechin-3-gallate [3, 56] may possess a therapeutic index in *IDH1*^MUT^ glioma.

## MATERIALS AND METHODS

### Patient selection

Gene expression analysis of LGG and glioblastoma data from TCGA (http://cancergenome.nih.gov) was conducted on processed data of the UCSC Cancer Genome Browser portal [63] and the cBioPortal for Cancer Genomics [64, 65]. The molecular data of 399 *IDH1*^MUT^ versus 112 *IDH1*^WT^ LGG and 157 *IDH1*^WT^ versus 9 *IDH1*^MUT^ glioblastoma samples were subsetted according to the *IDH1* mutational status and analyzed for expression and methylation of metabolic enzymes. Another 16 LGG and 198 glioblastoma tumors were excluded because of unknown *IDH1* mutational status or lack of molecular data.

### *In silico* analysis

Relative mRNA expression (RNAseq, IlluminaHiSeq) values were identified for relevant genes encoding for metabolic enzymes involved in glycolysis, the TCA cycle, glutamine-glutamate cycle and acetate cycle (Supplementary Table 1). Annotations were obtained from the Kyoto Encyclopedia of Genes and Genomes (KEGG). Mean normalized z-scores for mRNA levels were determined for *IDH1*^MUT^ and *IDH1*^WT^ datasets. The expression of each gene in each sample was normalized separately. The returned value indicated the number of standard deviations away from the population mean. In the methylation data, the probes with the strongest negative correlation between the methylation signal and gene expression were included.

### Cell culture

HCT116 *IDH1*^WT/R132H^ knock-in cells, generated by AAV targeting technology GENESIS (28), were kindly provided by Horizon Discovery (Cambridge, UK). *IDH1*^WT/R132H^ and *IDH1*^WT/WT^ HCT116 cells were cultured in McCoy's 5A medium (Gibco, Life Technologies, Thermo Fisher Scientific, Waltham, MA, USA) in 5% CO_2_ at 37°C. Media was supplemented with 10% fetal bovine serum (HyClone, Thermo Fisher Scientific), 100 units/ml penicillin and 100 μg/ml streptomycin (both Gibco).

### Reagents

AGI-5198 was purchased from MedChemExpress (Monmouth Junction, NJ, USA), olygomycin, carbonyl-cyanide-(trifluoromethoxy)phenylhydrazone (FCCP), antimycin A, rotenone, 2-deoxyglucose, glucose, L-glutamine and sodium pyruvate were purchased from Sigma-Aldrich. (St. Louis, MO, USA).

### Measurement of oxygen consumption rate and extracellular acidification rates

HCT 116 cells, with and without pre-treatment of 1μM AGI-5198, were grown in 5% CO_2_ at 37°C and OCR and ECAR was measured using an XFe96 analyzer (Seahorse Bioscience, North Billerica, MA, USA). HCT116 cells were plated in XF96 cell culture plates, 2.0*10^4^ *IDH1*^WT/R132H^ HCT116 cells or 1.75*10^4^ *IDH1*^WT/WT^ cells (to account for a more rapid proliferation of *IDH1*^WT/WT^ HCT116 cells relative to *IDH1*^WT/R132H^ HCT116 cells ([53] and our own observation), were seeded in each well of 96-well assay plates and incubated for 48 h prior to conducting the assay. For determination of OCR, medium was changed to DMEM supplemented with 10 mM glucose, 2 mM glutamine and 1.5 mM pyruvate. For the glycolytic flux, medium was changed to DMEM supplemented with 2 mM L-glutamine and the concentration of glucose added to initiate glycolysis was 25 mM. Four independent replicates were conducted with 10 technical replicates per cell line. Data were expressed as pmol of O_2_ per minute and normalized by cell number measured by the CyQUANT Cell proliferation kit (Invitrogen™), which is based on a fluorochrome binding to nucleic acids. Fluorescence was measured in a microplate luminometer (ClarioStar BMG Labtech, Cary, NC, USA) with excitation wavelength at 485 ± 10 nm and emission detection wavelength at 530 ± 12.5 nm.

### Statistical analysis

Statistical analysis included two-way Mann-Whitney tests to determine significance of expression differences observed between *IDH1*^MUT^ versus *IDH1*^WT^ samples and Kaplan–Meier estimates of survival with log-rank tests among strata. Relative expression data were visualized as box plots, depicting the 25^th^, 50^th^ and 75^th^ percentile, with 95% confidence intervals and significance levels are shown by (*P < 0.05), (**P < 0.01), (***P < 0.001) and (****P < 0.0001).

## SUPPLEMENTARY MATERIALS FIGURE AND TABLE



## References

[1] Chinot OL, Wick W, Mason W, Henriksson R, Saran F, Nishikawa R, Carpentier AF, Hoang-Xuan K, Kavan P, Cernea D, Brandes AA, Hilton M, Abrey L (2014). Bevacizumab plus radiotherapy-temozolomide for newly diagnosed glioblastoma. N Engl J Med.

[2] Cloughesy TF, Cavenee WK, Mischel PS (2014). Glioblastoma: from molecular pathology to targeted treatment. Annu Rev Pathol.

[3] Molenaar RJ, Botman D, Smits MA, Hira VV, van Lith SA, Stap J, Henneman P, Khurshed M, Lenting K, Mul AN, Dimitrakopoulou D, van Drunen CM, Hoebe RA (2015). Radioprotection of IDH1-mutated cancer cells by the IDH1-mutant inhibitor AGI-5198. Cancer Res.

[4] Parsons DW, Jones S, Zhang X, Lin JC, Leary RJ, Angenendt P, Mankoo P, Carter H, Siu IM, Gallia GL, Olivi A, McLendon R, Rasheed BA (2008). An integrated genomic analysis of human glioblastoma multiforme. Science.

[5] Balss J, Meyer J, Mueller W, Korshunov A, Hartmann C, von Deimling A (2008). Analysis of the IDH1 codon 132 mutation in brain tumors. Acta Neuropathol.

[6] Molenaar RJ, Radivoyevitch T, Maciejewski JP, van Noorden CJ, Bleeker FE (2014). The driver and passenger effects of isocitrate dehydrogenase 1 and 2 mutations in oncogenesis and survival prolongation. Biochim Biophys Acta.

[7] Xu W, Yang H, Liu Y, Yang Y, Wang P, Kim SH, Ito S, Yang C, Wang P, Xiao MT, Liu LX, Jiang WQ, Liu J (2011). Oncometabolite 2-hydroxyglutarate is a competitive inhibitor of alpha-ketoglutarate-dependent dioxygenases. Cancer Cell.

[8] Dang L, White DW, Gross S, Bennett BD, Bittinger MA, Driggers EM, Fantin VR, Jang HG, Jin S, Keenan MC, Marks KM, Prins RM, Ward PS (2009). Cancer-associated IDH1 mutations produce 2-hydroxyglutarate. Nature.

[9] He YF, Li BZ, Li Z, Liu P, Wang Y, Tang Q, Ding J, Jia Y, Chen Z, Li L, Sun Y, Li X, Dai Q (2011). Tet-mediated formation of 5-carboxylcytosine and its excision by TDG in mammalian DNA. Science.

[10] Figueroa ME, Abdel-Wahab O, Lu C, Ward PS, Patel J, Shih A, Li Y, Bhagwat N, Vasanthakumar A, Fernandez HF, Tallman MS, Sun Z, Wolniak K (2010). Leukemic IDH1 and IDH2 mutations result in a hypermethylation phenotype, disrupt TET2 function, and impair hematopoietic differentiation. Cancer Cell.

[11] Chowdhury R, Yeoh KK, Tian YM, Hillringhaus L, Bagg EA, Rose NR, Leung IK, Li XS, Woon EC, Yang M, McDonough MA, King ON, Clifton IJ (2011). The oncometabolite 2-hydroxyglutarate inhibits histone lysine demethylases. EMBO Rep.

[12] Kaelin WG (2011). Cancer and altered metabolism: potential importance of hypoxia-inducible factor and 2-oxoglutarate-dependent dioxygenases. Cold Spring Harb Symp Quant Biol.

[13] van Lith SA, Molenaar R, van Noorden CJ, Leenders WP (2014). Tumor cells in search for glutamate: an alternative explanation for increased invasiveness of IDH1 mutant gliomas. Neuro Oncol.

[14] Zhao S, Lin Y, Xu W, Jiang W, Zha Z, Wang P, Yu W, Li Z, Gong L, Peng Y, Ding J, Lei Q, Guan KL (2009). Glioma-derived mutations in IDH1 dominantly inhibit IDH1 catalytic activity and induce HIF-1alpha. Science.

[15] Koivunen P, Lee S, Duncan CG, Lopez G, Lu G, Ramkissoon S, Losman JA, Joensuu P, Bergmann U, Gross S, Travins J, Weiss S, Looper R (2012). Transformation by the (R)-enantiomer of 2-hydroxyglutarate linked to EGLN activation. Nature.

[16] Hanahan D, Weinberg RA (2011). Hallmarks of cancer: the next generation. Cell.

[17] Warburg O (1956). On the origin of cancer cells. Science.

[18] Vander Heiden MG, Cantley LC, Thompson CB (2009). Understanding the Warburg effect: the metabolic requirements of cell proliferation. Science.

[19] Lunt SY, Vander Heiden MG (2011). Aerobic glycolysis: meeting the metabolic requirements of cell proliferation. Annu Rev Cell Dev Biol.

[20] Oudard S, Arvelo F, Miccoli L, Apiou F, Dutrillaux AM, Poisson M, Dutrillaux B, Poupon MF (1996). High glycolysis in gliomas despite low hexokinase transcription and activity correlated to chromosome 10 loss. Br J Cancer.

[21] Fack F, Espedal H, Keunen O, Golebiewska A, Obad N, Harter PN, Mittelbronn M, Bahr O, Weyerbrock A, Stuhr L, Miletic H, Sakariassen PO, Stieber D (2015). Bevacizumab treatment induces metabolic adaptation toward anaerobic metabolism in glioblastomas. Acta Neuropathol.

[22] Talasila KM, Rosland GV, Hagland HR, Eskilsson E, Flones IH, Fritah S, Azuaje F, Atai N, Harter PN, Mittelbronn M, Andersen M, Joseph JV, Hossain JA (2016). The angiogenic switch leads to a metabolic shift in human glioblastoma. Neuro Oncol.

[23] Hamans B, Navis AC, Wright A, Wesseling P, Heerschap A, Leenders W (2013). Multivoxel (1)H MR spectroscopy is superior to contrast-enhanced MRI for response assessment after anti-angiogenic treatment of orthotopic human glioma xenografts and provides handles for metabolic targeting. Neuro Oncol.

[24] Molenaar RJ, Verbaan D, Lamba S, Zanon C, Jeuken JW, Boots-Sprenger SH, Wesseling P, Hulsebos TJ, Troost D, van Tilborg AA, Leenstra S, Vandertop WP, Bardelli A (2014). The combination of IDH1 mutations and MGMT methylation status predicts survival in glioblastoma better than either IDH1 or MGMT alone. Neuro Oncol.

[25] Bleeker FE, Atai NA, Lamba S, Jonker A, Rijkeboer D, Bosch KS, Tigchelaar W, Troost D, Vandertop WP, Bardelli A, Van Noorden CJ (2010). The prognostic IDH1(R132) mutation is associated with reduced NADP+-dependent IDH activity in glioblastoma. Acta Neuropathol.

[26] Miranda-Goncalves V, Honavar M, Pinheiro C, Martinho O, Pires MM, Pinheiro C, Cordeiro M, Bebiano G, Costa P, Palmeirim I, Reis RM, Baltazar F (2013). Monocarboxylate transporters (MCTs) in gliomas: expression and exploitation as therapeutic targets. Neuro Oncol.

[27] Parks SK, Chiche J, Pouyssegur J (2013). Disrupting proton dynamics and energy metabolism for cancer therapy. Nat Rev Cancer.

[28] van Lith SA, Navis AC, Verrijp K, Niclou SP, Bjerkvig R, Wesseling P, Tops B, Molenaar R, van Noorden CJ, Leenders WP (2014). Glutamate as chemotactic fuel for diffuse glioma cells: are they glutamate suckers?. Biochim Biophys Acta.

[29] Tonjes M, Barbus S, Park YJ, Wang W, Schlotter M, Lindroth AM, Pleier SV, Bai AH, Karra D, Piro RM, Felsberg J, Addington A, Lemke D (2013). BCAT1 promotes cell proliferation through amino acid catabolism in gliomas carrying wild-type IDH1. Nat Med.

[30] Moschen I, Broer A, Galic S, Lang F, Broer S (2012). Significance of short chain fatty acid transport by members of the monocarboxylate transporter family (MCT). Neurochem Res.

[31] Lenting K, Verhaak R, Ter Laan M, Wesseling P, Leenders W (2017). Glioma: experimental models and reality. Acta Neuropathol.

[32] Wu M, Neilson A, Swift AL, Moran R, Tamagnine J, Parslow D, Armistead S, Lemire K, Orrell J, Teich J, Chomicz S, Ferrick DA (2007). Multiparameter metabolic analysis reveals a close link between attenuated mitochondrial bioenergetic function and enhanced glycolysis dependency in human tumor cells. Am J Physiol Cell Physiol.

[33] Chan DA, Sutphin PD, Nguyen P, Turcotte S, Lai EW, Banh A, Reynolds GE, Chi JT, Wu J, Solow-Cordero DE, Bonnet M, Flanagan JU, Bouley DM (2011). Targeting GLUT1 and the Warburg effect in renal cell carcinoma by chemical synthetic lethality. Sci Transl Med.

[34] Zhang J, Nuebel E, Wisidagama DR, Setoguchi K, Hong JS, Van Horn CM, Imam SS, Vergnes L, Malone CS, Koehler CM, Teitell MA (2012). Measuring energy metabolism in cultured cells, including human pluripotent stem cells and differentiated cells. Nat Protoc.

[35] Gatenby RA, Gillies RJ (2004). Why do cancers have high aerobic glycolysis?. Nat Rev Cancer.

[36] Walenta S, Mueller-Klieser WF (2004). Lactate: mirror and motor of tumor malignancy. Semin Radiat Oncol.

[37] Mazurek S, Boschek CB, Hugo F, Eigenbrodt E (2005). Pyruvate kinase type M2 and its role in tumor growth and spreading. Semin Cancer Biol.

[38] Lunt SY, Muralidhar V, Hosios AM, Israelsen WJ, Gui DY, Newhouse L, Ogrodzinski M, Hecht V, Xu K, Acevedo PN, Hollern DP, Bellinger G, Dayton TL (2015). Pyruvate kinase isoform expression alters nucleotide synthesis to impact cell proliferation. Mol Cell.

[39] Christofk HR, Vander Heiden MG, Harris MH, Ramanathan A, Gerszten RE, Wei R, Fleming MD, Schreiber SL, Cantley LC (2008). The M2 splice isoform of pyruvate kinase is important for cancer metabolism and tumour growth. Nature.

[40] Anastasiou D, Poulogiannis G, Asara JM, Boxer MB, Jiang JK, Shen M, Bellinger G, Sasaki AT, Locasale JW, Auld DS, Thomas CJ, Vander Heiden MG, Cantley LC (2011). Inhibition of pyruvate kinase M2 by reactive oxygen species contributes to cellular antioxidant responses. Science.

[41] Wolf A, Agnihotri S, Micallef J, Mukherjee J, Sabha N, Cairns R, Hawkins C, Guha A (2011). Hexokinase 2 is a key mediator of aerobic glycolysis and promotes tumor growth in human glioblastoma multiforme. J Exp Med.

[42] Izquierdo-Garcia JL, Viswanath P, Eriksson P, Chaumeil MM, Pieper RO, Phillips JJ, Ronen SM (2015). Metabolic reprogramming in mutant IDH1 glioma cells. PLoS One.

[43] Chesnelong C, Chaumeil MM, Blough MD, Al-Najjar M, Stechishin OD, Chan JA, Pieper RO, Ronen SM, Weiss S, Luchman HA, Cairncross JG (2014). Lactate dehydrogenase A silencing in IDH mutant gliomas. Neuro Oncol.

[44] Chaumeil MM, Lupo JM, Ronen SM (2015). Magnetic Resonance (MR) Metabolic imaging in glioma. Brain Pathol.

[45] Mustafa DA, Swagemakers SM, Buise L, van der Spek PJ, Kros JM (2014). Metabolic alterations due to IDH1 mutation in glioma: opening for therapeutic opportunities?. Acta Neuropathol Commun.

[46] Ho J, de Moura MB, Lin Y, Vincent G, Thorne S, Duncan LM, Hui-Min L, Kirkwood JM, Becker D, Van Houten B, Moschos SJ (2012). Importance of glycolysis and oxidative phosphorylation in advanced melanoma. Mol Cancer.

[47] McCleland ML, Adler AS, Deming L, Cosino E, Lee L, Blackwood EM, Solon M, Tao J, Li L, Shames D, Jackson E, Forrest WF, Firestein R (2013). Lactate dehydrogenase B is required for the growth of KRAS-dependent lung adenocarcinomas. Clin Cancer Res.

[48] Mirebeau-Prunier D, Le Pennec S, Jacques C, Fontaine JF, Gueguen N, Boutet-Bouzamondo N, Donnart A, Malthiery Y, Savagner F (2013). Estrogen-related receptor alpha modulates lactate dehydrogenase activity in thyroid tumors. PLoS One.

[49] Baldock AL, Yagle K, Born DE, Ahn S, Trister AD, Neal M, Johnston SK, Bridge CA, Basanta D, Scott J, Malone H, Sonabend AM, Canoll P (2014). Invasion and proliferation kinetics in enhancing gliomas predict IDH1 mutation status. Neuro Oncol.

[50] Cuyas E, Fernandez-Arroyo S, Corominas-Faja B, Rodriguez-Gallego E, Bosch-Barrera J, Martin-Castillo B, De Llorens R, Joven J, Menendez JA (2015). Oncometabolic mutation IDH1 R132H confers a metformin-hypersensitive phenotype. Oncotarget.

[51] Li F, He X, Ye D, Lin Y, Yu H, Yao C, Huang L, Zhang J, Wang F, Xu S, Wu X, Liu L, Yang C (2015). NADP(+)-IDH Mutations promote hypersuccinylation that impairs mitochondria respiration and induces apoptosis resistance. Mol Cell.

[52] Sjoblom T, Jones S, Wood LD, Parsons DW, Lin J, Barber TD, Mandelker D, Leary RJ, Ptak J, Silliman N, Szabo S, Buckhaults P, Farrell C (2006). The consensus coding sequences of human breast and colorectal cancers. Science.

[53] Grassian AR, Parker SJ, Davidson SM, Divakaruni AS, Green CR, Zhang X, Slocum KL, Pu M, Lin F, Vickers C, Joud-Caldwell C, Chung F, Yin H (2014). IDH1 mutations alter citric acid cycle metabolism and increase dependence on oxidative mitochondrial metabolism. Cancer Res.

[54] Izquierdo-Garcia JL, Viswanath P, Eriksson P, Cai L, Radoul M, Chaumeil MM, Blough M, Luchman HA, Weiss S, Cairncross JG, Phillips JJ, Pieper RO, Ronen SM (2015). IDH1 Mutation induces reprogramming of pyruvate metabolism. Cancer Res.

[55] Mashimo T, Pichumani K, Vemireddy V, Hatanpaa KJ, Singh DK, Sirasanagandla S, Nannepaga S, Piccirillo SG, Kovacs Z, Foong C, Huang Z, Barnett S, Mickey BE (2014). Acetate is a bioenergetic substrate for human glioblastoma and brain metastases. Cell.

[56] Zhang J, Wang G, Mao Q, Li S, Xiong W, Lin Y, Ge J (2016). Glutamate dehydrogenase (GDH) regulates bioenergetics and redox homeostasis in human glioma. Oncotarget.

[57] Chaumeil MM, Larson PE, Woods SM, Cai L, Eriksson P, Robinson AE, Lupo JM, Vigneron DB, Nelson SJ, Pieper RO, Phillips JJ, Ronen SM (2014). Hyperpolarized [1-13C] glutamate: a metabolic imaging biomarker of IDH1 mutational status in glioma. Cancer Res.

[58] Izquierdo-Garcia JL, Cai LM, Chaumeil MM, Eriksson P, Robinson AE, Pieper RO, Phillips JJ, Ronen SM (2014). Glioma cells with the IDH1 mutation modulate metabolic fractional flux through pyruvate carboxylase. PLoS One.

[59] Brat DJ, Verhaak RG, Aldape KD, Yung WK, Salama SR, Cooper LA, Rheinbay E, Miller CR, Vitucci M, Morozova O, Robertson AG, Noushmehr H, Cancer Genome Atlas Research N (2015). Comprehensive, integrative genomic analysis of diffuse lower-grade gliomas. N Engl J Med.

[60] Feichtinger RG, Weis S, Mayr JA, Zimmermann F, Geilberger R, Sperl W, Kofler B (2014). Alterations of oxidative phosphorylation complexes in astrocytomas. Glia.

[61] Vander Heiden MG, Christofk HR, Schuman E, Subtelny AO, Sharfi H, Harlow EE, Xian J, Cantley LC (2010). Identification of small molecule inhibitors of pyruvate kinase M2. Biochem Pharmacol.

[62] Vartanian A, Agnihotri S, Wilson MR, Burrell KE, Tonge PD, Alamsahebpour A, Jalali S, Taconne MS, Golbourn B, Aldape KD, Zadeh G (2016). Targeting hexokinase 2 enhances response to radio-chemotherapy in glioblastoma. Oncotarget.

[63] Zhu J, Sanborn JZ, Benz S, Szeto C, Hsu F, Kuhn RM, Karolchik D, Archie J, Lenburg ME, Esserman LJ, Kent WJ, Haussler D, Wang T (2009). The UCSC Cancer Genomics Browser. Nat Methods.

[64] Cerami E, Gao J, Dogrusoz U, Gross BE, Sumer SO, Aksoy BA, Jacobsen A, Byrne CJ, Heuer ML, Larsson E, Antipin Y, Reva B, Goldberg AP (2012). The cBio cancer genomics portal: an open platform for exploring multidimensional cancer genomics data. Cancer Discov.

[65] Gao J, Aksoy BA, Dogrusoz U, Dresdner G, Gross B, Sumer SO, Sun Y, Jacobsen A, Sinha R, Larsson E, Cerami E, Sander C, Schultz N (2013). Integrative analysis of complex cancer genomics and clinical profiles using the cBioPortal. Sci Signal.

