# Supplementary material for: In silico gene expression analysis reveals glycolysis and acetate anaplerosis in IDH1 wild-type glioma and lactate and glutamate anaplerosis in IDH1-mutated glioma
Source: Oncotarget. 2017 Apr 13;8(30):49165–77. doi: 10.18632/oncotarget.17106 (PMC5564758; doi:10.18632/oncotarget.17106)
Supplement: Supplementary file 1 [file oncotarget-08-49165-s001.pdf]

In silico gene expression analysis reveals glycolysis and acetate anaplerosis in *IDH1* wild-type glioma and lactate and glutamate anaplerosis in *IDH1*-mutated glioma

Supplementary Materials

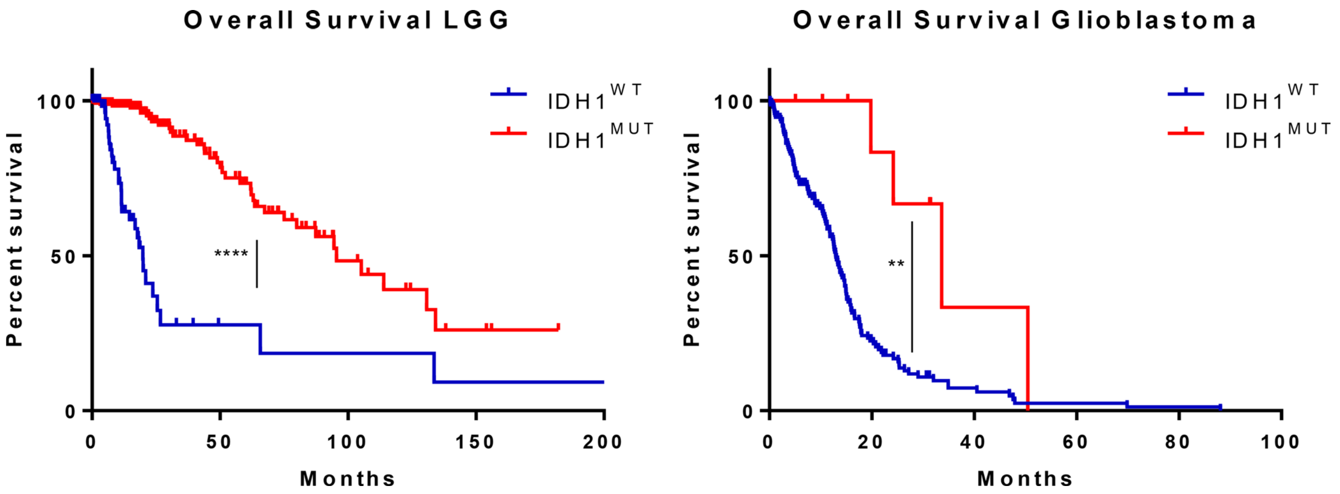

Supplementary Figure 1: Survival curves comparing *IDH1*^{MUT} and *IDH1*^{WT} in LGG and glioblastoma patients in our created dataset. 399 *IDH1*^{MUT} versus 112 *IDH1*^{WT} LGG and 157 *IDH1*^{WT} versus 9 *IDH1*^{MUT} glioblastoma patients. Kaplan–Meier estimates of survival with log-rank tests among strata.

Supplementary Table 1: Selection of genes encoding for enzymes of the glycolytic pathway, TCA cycle, glutamine-glutamate cycle and acetate cycle

Enzyme	Gene Symbol	GeneID
**Glucose transporter**	SLC2A1	6513
	SLC2A2	6514
	SLC2A3	6515
**Hexokinase**	HK1	3098
	HK2	3099
	HK3	3101
**Glucose-6-phosphate dehydrogenase**	G6PD	2539
**Pyruvate kinase**	PKLR	5313
	PKM2	5315
**Lactate dehydrogenase**	LDHA	3939
	LDHB	3945
**Monocarboxylacte transporter**	SLC16A1	6566
	SLC16A3	9123
**Pyruvate dehydrogenase**	PDHA1	5160
	PDHA2	5161
	PDHB	5162
	PDHX	8050
**Pyruvate dehydrogenase kinase**	PDK1	5163
	PDK2	5164
	PDK3	5165
**Citrate synthase**	CS	1431
**Aconitase**	ACO2	50
	ACO1	48
**Isocitrate dehydrogenase**	IDH1	3417
	IDH2	3418
	IDH3B	3420
	IDH3G	3421
	IDH3A	3419
**α-Ketoglutarate dehydrogenase**	OGDH	4967
**Succinate dehydrogenase**	SDHA	6389
	SDHB	6390
	SDHC	6391
	SDHD	6392
**Fumarate hydratase**	FH	2271
**Malate dehydrogenase**	MDH1	4190
	MDH2	4191
**Pyruvate carboxylase**	PC	5091
**Phosphoenolpyruvate carboxykinase**	PCK1	5105
	PCK2	5106
**Acyl-CoA synthetase**	ACSS1	84532
	ACSS2	55902
**Glutamate dehydrogenase**	GLUD1	2746
	GLUD2	2747
**Branched chain amino acid transaminase**	BCAT1	586
	BCAT2	587
**Glutaminase**	GLS	2744
	GLS2	27165
**Glutamate-ammonia ligase**	GLUL	2752

Gene symbol and GeneID were obtained from the Kyoto Encyclopedia of Genes and Genomes.
